# Attachment Representation Moderates the Effectiveness of Behavioral Parent Training Techniques for Children with ADHD: Evidence from a Randomized Controlled Microtrial

**DOI:** 10.1007/s10802-022-00921-5

**Published:** 2022-04-01

**Authors:** Rianne Hornstra, Tycho J. Dekkers, Guy Bosmans, Barbara van den Hoofdakker, Saskia van der Oord

**Affiliations:** 1 Department of Child and Adolescent Psychiatry, University Medical Center Groningen, University of Groningen, Groningen, the Netherlands; 2 Accare Child Study Center, Groningen, the Netherlands; 3 Academic Center for Child and Adolescent Psychiatry, Levvel, Amsterdam, the Netherlands; 4 Department of Psychology, University of Amsterdam, Amsterdam, the Netherlands; 5 Department of Child and Adolescent Psychiatry, Amsterdam University Medical Center (AUMC), Amsterdam, the Netherlands; 6 Department of Clinical Psychology and Experimental Psychopathology, University of Groningen, Groningen, the Netherlands; 7 Department of Clinical Psychology, KU Leuven, Leuven, Belgium; 8 Leuven Brain Institute, Leuven, Belgium

**Keywords:** Attention-Deficit, Hyperactivity Disorder (ADHD), Attachment, Children, Behavioral parent training, Techniques, Story stem

## Abstract

Behavioral parent training is an evidence-based intervention for children with attention-deficit/hyperactivity disorder (ADHD), but knowledge on the differential effects of behavioral techniques for specific subgroups of children is very limited. Attachment representations of children with ADHD may affect how receptive children are to changes in parenting. In this study, we investigated whether specific behavioral techniques were more or less effective for children with ADHD in relation to their attachment representations. We included parents of 74 children with ADHD (4–11 years, *M* = 8.15) who took part in a larger randomized controlled microtrial in which they were randomized to a two session training in antecedent-based techniques (i.e., stimulus control techniques: rules, instructions; *n* = 26), a two session training in consequent-based techniques (i.e., contingency management techniques: praise, rewards, ignoring; *n* = 25) or a waitlist control condition (*n* = 23). We examined whether attachment representation moderated the effectiveness of a) training versus waitlist, and b) antecedent- versus consequent-based techniques. Attachment representations were measured with a story stem task, the intervention outcome was daily parent-rated problem behaviors of the children. Attachment representation did not moderate the effects of the training compared to the waitlist. However, compared to antecedent-based techniques, consequent-based techniques were less effective for more securely and less disorganized attached children, and particularly effective for more disorganized attached children. This was the first study examining attachment as a moderator of behavioral techniques for ADHD. If replicated, the findings of this study can be used for treatment development and tailoring.

**Supplementary Information:**

The online version contains supplementary material available at 10.1007/s10802-022-00921-5.

## Introduction

Attention-deficit/hyperactivity disorder (ADHD) is characterized by impairing symptoms of inattention, hyperactivity and/or impulsivity (DSM-5; American Psychiatric Association, 2013), and is one of the most commonly diagnosed childhood psychiatric disorders worldwide (Antshel, 2015; Polanczyk et al., 2015). Evidence-based treatment for ADHD includes both pharmacological and psychosocial interventions, with behavioral parent training (BPT) as the primary, most studied psychosocial intervention for school-age children with ADHD (Daley et al., 2018; Dekkers et al., 2021a; Evans et al., 2018).

BPT has a social learning theoretical foundation, and includes principles of operant/instrumental conditioning (Shaffer et al., 2001). Parents are being trained in techniques that are aimed at changing behavior by increasing the discriminative value of stimuli predicting adaptive/desired behavior (antecedent-based, or stimulus control techniques), or techniques to change contingencies, thus reinforce desired behaviors, and ignore or punish undesired behaviors (consequent-based, or contingency management techniques) (Antshel, 2015). Antecedent-based techniques include providing clear instructions and structure to clarify what behavior is expected in specific situations. Consequent-based techniques are for example the use of praise, ignoring or mild punishment (Antshel & Barkley, 2008). Most, if not all, interventions combine both types of techniques, with an emphasis on either antecedent-based or consequent-based techniques (Dekkers et al., 2021a).

Meta-analytic evidence supports the effectiveness of BPT as a treatment for children with ADHD, but effect sizes of these programs are modest at best and not all individuals benefit equally (Coates et al., 2015; Fabiano et al., 2015; Lee et al., 2012). One way to improve behavioral parent training programs may be to disentangle which components make these programs effective, and to identify subgroups of children for whom these components are more or less effective (Leijten et al., 2021). However, knowledge on the effectiveness of the separate behavioral techniques that make up BPT is very limited, as programs are usually examined as a whole. Therefore, we recently conducted a randomized controlled microtrial, in which we compared two sessions of antecedent-based techniques or consequent-based techniques to a waitlist control condition on daily parent-rated problem behaviors of children with ADHD. Both types of techniques were effective as compared to the control condition, with moderate effect sizes and no significant differences between antecedent- and consequent-based techniques (Hornstra et al., 2021). To further guide the personalization of BPT, research focusing on the effectiveness of behavioral techniques for specific subgroups of children is imperative (Ng & Weisz, 2016). Therefore, in the current study, we further disentangle the findings of our microtrial by investigating potential moderating effects of attachment representations on the outcomes of separate techniques of BPT. This can ultimately lead to evidence-based personalization of complete interventions to meet families’ specific needs (Leijten et al., 2015).

Randomized controlled microtrials are ideal to examine specific moderators of effectiveness of particular isolated techniques for a number of reasons. First, in a microtrial, only one component of an intervention (instead of a full program) is tested in isolation, therefore minimizing the influence of other components or factors. Second, the component is carried out in a controlled and optimal way, potentially creating stronger manipulations. Third, microtrials examine effects on proximal child behavior in the here and now (i.e., a proximal, specific outcome), closest to the manipulation. As other influences potentially add noise over time, these outcome measures allow for the detection of effects that would otherwise become harder to identify. Due to these advantages, experimental microtrial designs have more power to detect potential moderation effects (Howe et al., 2010; Howe & Ridenour, 2019). In a previous paper we examined the influence of several child characteristics (i.e., age, IQ, sex, parental education level, baseline levels of ADHD, ODD, and CD symptoms, and impairment) on the effectiveness of these separate techniques. We found no moderating effects of these variables: antecedent- and consequent-based techniques were equally effective for the subgroups of children identified by these variables (Hornstra et al., 2021).

In the current study, we further investigate whether specific techniques are more or less effective for certain subgroups of children with ADHD, using attachment as a potential moderator. To date, although relevant, in the broader behavioral problems literature, studies examining whether children with a certain attachment representation respond differentially to specific behavioral techniques is absent. Research shows that differences in sensitive parenting are associated with differences in children’s attachment development (Beijersbergen et al., 2012; De Wolff & van Ijzendoorn, 1997). If a caregiver reacts sensitively and supportive towards distress signals from the child it is believed that the child will form an internal working model of the attachment figure as competent and predictable, whose proximity and safety can be solicited during exploration of the environment (Waters & Waters, 2006). According to attachment theory, this results in the development of a secure attachment to that figure (Bowlby, 1980). Insecure attachment, on the other hand, reflects a lack of trust in the availability of the caregiver when the child needs protection or support, leading to high levels of ambivalent attachment, avoidant attachment, or both. Ambivalent attachment represents the tendency to focus attention exclusively towards the caregiver. Ambivalent attached children often react with significant distress if they are separated from their caregivers and seek close proximity towards them. Avoidant attached children tend to cope with stressful situations by redirecting their attention away from the caregiver and are characterized by excessive self-reliance. Lastly, children with a disorganized attachment show disoriented or contradictory behaviors, not representing a coherent attachment strategy (Main & Cassidy, 1988).

The learning theory of attachment (Bosmans et al., 2020) proposes that attachment is embedded in the genetic make-up of children, and its development is subject to classical and operant learning principles. Attachment development can be interpreted as a safety conditioning process in which the caregiver can become a safety cue; a predictor that an aversive event will not follow. It has been suggested that certain child characteristics can influence attachment development through differences in neurobiological preparedness for attachment-safety conditioning, making it harder for caregivers to support secure attachment development in their child. In children with ADHD, proposed underlying causes and deficits of ADHD may have implications for their susceptibility to parenting practices (Van der Oord & Tripp, 2020). Due to attentional difficulties, it may be challenging for these children to notice (changes in) sensitive parenting. Also, motivational problems, such as altered reward and punishment sensitivity, can complicate learning from parenting behaviors. Indeed, an increasing number of studies demonstrate an association between insecure or disorganized attachment styles and ADHD (Wylock et al., 2021), differences between children with ADHD and typically developing children in attachment representations (ADHD less secure/ more disorganized) (Dekkers et al., 2021b), and a recent meta-analysis reported a significant link between attention problems and insecure and disorganized attachment styles (Pallini et al., 2019). Taken together, it could be that children with ADHD respond differently to (changes in) parenting, due to neurobiological difficulties related to (safety) conditioning (Tripp & Wickens, 2008). Therefore, due to altered reward processing and attentional problems specific to ADHD, attachment representation of a child could potentially influence attachment development and the effectiveness of behavioral techniques used by parents.

The current study had two main aims. First, we investigated the influence of attachment representations of children with ADHD on the effectiveness of a brief (i.e., two-session) BPT, which consisted of either antecedent- or consequent-based techniques (relative to a waitlist control condition). To our knowledge, research into children’s attachment representation as a predictor of treatment outcome for BPT, especially in children with ADHD, is nonexistent. Overall, in psychotherapy, securely attached individuals have better therapy outcomes relative to insecurely attached individuals (see Levy et al., 2018; Slade & Holmes, 2019 for meta-analytic reviews). In children with conduct problems, a better parent–child relationship (which is related to secure attachment) was positively linked to better outcomes of BPT (Dedousis-Wallace et al., 2021). Likewise, attachment security could improve treatment success of BPT for children with ADHD. Moreover, children who are less securely attached can have automatic information-processing biases (a more negative interpretation of attachment-related information) which can limit the effects of newly learned strategies by parents in BPT (Verhees et al., 2019). Therefore, a lack of trust of the child in the caregivers potentially reduces the likelihood that improved parenting leads to a better parent–child relationship and improvement in behaviors of the child (Bosmans, 2016). We thus hypothesized that higher levels of secure and lower levels of insecure and disorganized child-parent attachment representations would positively affect treatment success of BPT (for which both AC and CC were taken together into one ‘training’ condition) compared to a waitlist; and that lower levels of secure and higher levels of insecure and disorganized child-parent attachment would negatively affect treatment success.

Our second aim was to investigate whether children’s attachment representation moderated the effectiveness of the antecedent-based techniques compared to the consequent-based techniques. Because of the scarcity of studies examining moderators of separate behavioral techniques on treatment response, the second part of the study was rather exploratory and hypothesis-generating. We speculated that in children with ADHD particularly consequent-based techniques (e.g., rewards, discipline strategies) can be differentially effective depending on the attachment representation of the child. Especially in parent–child dyads where the child is insecurely attached, learnt rewarding strategies provided by the parent can be interpreted by the child as insincere, and discipline strategies can be interpreted ambivalent and hostile (Dadds & Hawes, 2006). For example, insecure attachment of the child can decrease the perceived value of rewards given by the parents (e.g., praise the child for desired behavior), and can trigger disturbed attachment-seeking behaviors of the child when a disciplining strategy by the parent (e.g., a correction for undesired behavior) is applied (Owen et al., 2012). Above this, motivational problems in children with ADHD (i.e., altered reward and punishment sensitivity), can further complicate learning from these consequent-based techniques (Van der Oord & Tripp, 2020). Changes in sensitive parenting may be more difficult to assimilate and children may need more time to adjust to these changes. Thus, we hypothesized that children with ADHD who are insecurely or disorganized attached to their caregiver would respond less favorable to consequent-based techniques. Antecedent-based techniques (e.g., stating clear rules and instructions) on the other hand, are being used preventatively and prior to behavioral problems, and thus prevent potential troublesome interactions between the parent and child (Kalb & Loeber, 2003). Therefore, we speculated that attachment representations of children would have less of an influence on the effectiveness of the antecedent-based techniques.

## Method

### Participants

The current sample comprised 74 children (age 4–12) whose parents participated in a randomized controlled microtrial (Hornstra et al., 2021, *N* = 92) in which we investigated effects of antecedent-based and consequent-based techniques. Data from 18 children of the original sample were missing because of technical problems. Characteristics of the current subsample of children and their parents are presented in Table 1. The inclusion criteria for the study were (1) a Diagnostic and Statistical Manual of Mental Disorders-5 (DSM-5) based classification of ADHD, confirmed with the Diagnostic Interview Schedule for Children-IV, parent interview (DISC-IV; Shaffer et al., 2000), which we adapted to the DSM-5; (2) at least four parent-rated problem behaviors, as assessed with extensive list of 29 different inattentive, hyperactive, impulsive and oppositional defiant behaviors (Hornstra et al., 2021; Van den Hoofdakker et al., 2007) scored as three or higher on a five-point Likert scale, ranging from 1 (not severe) to 5 (extremely severe); (3) the child having an IQ > 70. This was derived from the patient file, or if not present measured with two subtests (‘Vocabulary’ and ‘Block design’; Sattler, 2008) of the Wechsler Intelligence Scale for Children (WISC-III-NL) or, if the child was younger than six years old, the Wechsler Preschool and Primary Scale of Intelligence (WPSSI-III-NL); (4) no current psychotropic medication use. If the child previously used psychotropic medication, he/she had to be off medication at least 4 weeks before the start of the study. Exclusion criteria were (1) the child having a diagnosis of autism spectrum disorder (as reported by the parent or derived from the patient file) or conduct disorder (derived from the adapted DISC-IV (Shaffer et al., 2000) or the patient file), because these children might have more intensive treatment needs than a two session experimental treatment, and to examine the effects of the separate techniques in a fairly homogeneous group, (2) the caregivers received BPT in the past year, and (3) the child was not living in the same house during the weekdays (because our primary outcome measure had to be reported by the same caregiver).

**Table 1:** Characteristics of the children and their parents per condition

	AC (*n* = 26)	CC (*n* = 25)	WL (*n* = 23)	Group comparisons	*p*
Age in years, M (SD)	8.0 (1.7)	8.4 (1.8)	8.00 (1.6)	*F*(2,71) = 0.57	0.57
IQ, M (SD)	94.7 (12.9)	100.9 (14.5)	92.5 (11.2)	*F*(2,71) = 2.76	0.07
Sex, *n* (%) boys	18 (69.2)	16 (64.0)	14 (60.9)	*χ*^2^(2) = 0.39	0.82
Parental education level, *n* (%) ^a^					
Low	2 (7.7)	6 (24.0)	5 (21.7)	*χ*^2^(2) = 2.88	0.24
Medium	10 (38.5)	7 (28.0)	8 (34.8)	*χ*^2^(2) = 0.70	0.71
High	13 (50.0)	11 (44.0)	8 (34.8)	*χ*^2^(2) = 0.89	0.64
Other psychiatric diagnoses, *n* (%)					
ODD ^b^	13 (50.0)	6 (24.0)	9 (39.1)	*χ*^2^(2) = 3.69	0.16
Learning disorder ^c^	1 (3.8)	1 (4.0)	1 (4.3)	*χ*^2^(2) = 0.01	1.00
Anxiety disorder ^c^	0 (0.0)	1 (4.0)	0 (0)	*χ*^2^(2) = 1.99	0.37
ADHD presentation, *n* (%) ^b^					
Combined	17 (65.4)	12 (48.0)	15 (65.2)	*χ*^2^(2) = 2.06	0.36
Predominantly inattentive	7 (26.9)	11 (44.0)	4 (17.4)	*χ*^2^(2) = 4.21	0.12
Predominantly hyperactive-impulsive	2 (7.7)	2 (8.0)	4 (17.4)	*χ*^2^(2) = 1.50	0.47
Number of symptoms, M (SD) ^c^					
Inattention symptoms	7.1 (1.4)	7.2 (1.3)	6.9 (1.4)	*F*(2,71) = 0.28	0.75
Hyperactivity-impulsivity symptoms	6.7 (2.1)	5.3 (2.3)	6.7 (1.9)	*F*(2,71) = 0.37	0.29
ODD symptoms	3.3 (2.2)	2.7 (1.9)	2.8 (2.3)	*F*(2,45.559) = 0.58^d^	0.56
CD symptoms	0.6 (1.2)	0.3 (0.5)	0.2 (0.6)	*F*(2,69) = 1.56	0.22
Impairment, M (SD) ^e^					
Number of impaired domains	3.9 (1.2)	3.4 (1.5)	3.7 (1.5)	*F*(2,68) = 1.04	0.36
Average score	6.1 (1.7)	5.6 (1.6)	5.8 (1.8)	*F*(2,68) = 1.86	0.52
Mean score of daily rated problem behaviors, M (SD)	2.5 (0.9)	2.2 (0.8)	2.3 (0.8)	*F*(2,71) = 0.74	0.48
Attachment representations, M (SD) ^f^					
Secure	2.9 (1.2)	2.7 (1.2)	2.6 (1.2)	*F*(2,71) = 0.53	0.59
Avoidant	1.8 (0.8)	2.0 (0.9)	1.9 (0.9)	*F*(2,71) = 0.32	0.73
Ambivalent	1.4 (0.7)	1.5 (0.6)	1.5 (0.6)	*F*(2,71) = 0.07	0.94
Disorganized	1.8 (1.2)	1.8 (1.1)	1.9 (1.1)	*F*(2,71) = 0.10	0.91

*AC* antecedent-based condition, *CC* consequent-based condition, *WL* Waitlist, *ADHD* attention-deficit/hyperactivity disorder, *ODD* oppositional defiant disorder, *CD* conduct disorder

^a^Parental education level (average of both parents) was classified according to the Dutch classification system: 1 = no education completed, 2 = early childhood education, 3 = primary education, 4 = lower secondary education, 5 = upper secondary education, 6 = undergraduate school, 7 = graduate school, 8 = post-graduate education, which was divided in low = 1, 2, 3, 4, medium = 5, and high = 6, 7, 8 (CBS, 2016)

^b^Assessed with the Diagnostic Interview Schedule for Children-IV-TR, adapted to the DSM-5

^c^Derived from the patient file

^d^If variances were unequal, Welch’s F-test was used instead of regular *F* tests

^e^Assessed with the Impairment Rating Scale; domains with a score > 3 were classified as impaired

^f^Assessed with the story stem procedure

### Procedure

This study was registered at the Dutch Trial Register: https://www.trialregister.nl/trial/6011. Medical ethical approval of the microtrial was waived by the Medical Ethical Committee of the University Medical Center Groningen (UMCG; METc 2016/197). After obtaining written informed consent, parents were randomized (using a random number generator, ratio of 1:1:1) to one of the two intervention conditions (i.e., antecedent- or consequent-based), or a waitlist control condition. Attachment representation was assessed prior to randomization. Outcome measures were assessed at three time points: prior to randomization (T0), during the week immediately after the intervention or the waiting period (T1), and three weeks after the intervention or waiting period (T2). After T2, all parents could receive care as usual. Parents randomly assigned to the waitlist control condition could receive one or both of the intervention conditions of choice of the current study at T2 as well. Since there were no guidelines for reporting on microtrials, we used the CONSORT-SPI 2018 extension (an extension of the main Consort statement; Montgomery et al., 2018) for reporting on randomized controlled trials of social and psychological interventions. The CONSORT-SPI 2018 differs from the standard CONSORT method as it extends 9 of the 25 items including background and objectives, trial design, participants, interventions, statistical methods, participant flow, baseline data, outcomes and estimation, and funding. Parents received a small compensation (€10) for participating in the study. For more details concerning the design of the study, see Hornstra et al. (2021).

### Interventions

There were two intervention conditions, one in which parents were trained in antecedent-based techniques (antecedent-based condition), and one in which parents were trained in consequent-based techniques (consequent-based condition). Both interventions were delivered individually and both caregivers (when applicable) were encouraged to engage in the sessions. The primary caregiver (i.e., the caregiver that spends most time with the child) always participated in the sessions. Each intervention condition consisted of two sessions (two hours each), and was provided if possible, in two consecutive weeks, by experienced psychologists who had comprehensive experience with ADHD and parent training and completed postgraduate training in behavior therapy. Both interventions were targeted to preselected problem behaviors from a list of 29 ADHD-related behaviors (including inattentive, hyperactive, and impulsive symptoms, and oppositional defiant behaviors; Hornstra et al., 2021; Van den Hoofdakker et al., 2007) in a specific situation (using the Home Situations Questionnaire; Breen & Altepeter, 1991) (see “Outcome Measure”).

The first session consisted of the following steps: (1) psycho-education about ADHD with specific information about a) how stimuli elicit behaviors, how executive functioning problems in children with ADHD may lead to problems in adapting their behavior to stimuli, and how antecedent-based techniques can be used to elicit desired behavior and prevent undesired behavior (i.e., antecedent-based condition), or b) how consequences influence behavior, how altered reward sensitivity in children with ADHD may cause problems in how their behavior is influenced by the environment, and how consequent-based techniques can support them by changing the consequences of behavior (i.e., consequent-based condition) (Van der Oord & Tripp, 2020); (2) the selection of one of the problem behaviors in a specific situation, based on frequency, severity, changeability, and burden to parents; (3) a detailed behavioral analysis of the selected behavior, and defining a desired target behavior, together with the parents; (4) a tailored intervention plan, consisting of a) antecedent-based techniques such as defining rules, giving clear instructions, anticipating misbehaviors, and providing routines, or b) consequent-based techniques such as planned ignoring, praise, rewards, and punishment, based on the function of the behavior (Virués-Ortega & Haynes, 2005); (5) practicing with the intervention plan, either through roleplay or visualization; (6) implementing the intervention plan by the parents at home for one week, after which the second session took place. The second session started with an evaluation of the previous week and, if necessary, adapting the intervention plan from the first session. After that, steps two to five were repeated for a different behavior in a specific situation. A more detailed description of the interventions can be found elsewhere (Hornstra et al., 2021).

### Measures

#### Outcome Measure

At T0, caregivers selected four behaviors from an extensive list of 29 different problem behaviors (Hornstra et al., 2021; Van den Hoofdakker et al., 2007) that they wanted to target during the sessions, and indicated in which situations the behaviors occurred most prominently (using the Home Situations Questionnaire; Breen & Altepeter, 1991). In short daily phone calls, made by RH or a research-assistant, the primary caregiver was asked to rate the severity of these behaviors on a scale from 0–5 (with 0 indicating the behavior did not occur, and 5 indicating that the behavior was extremely severe that day). A weekly mean-level score was calculated with the daily ratings of the four behaviors over five consecutive weekdays (a minimum of four days), except for holidays. Weekend-days were excluded because children tend to behave differently on week-days than on weekend-days, parents usually do not experience the selected problem behaviors on weekend-days, or the selected specific situations do not occur in weekends. The weekly mean-level score was calculated for five days on T0, T1, and T2. Reliability of this list of 29 problem behaviors in the current sample was excellent (α = 0.91). The daily ratings of the four behaviors can be seen of as an ecological momentary assessment (EMA) (Shiffman et al., 2008). EMA concerns repeated assessment of behavior in real time in the natural environment of participants, therefore reducing recall bias and increasing ecological validity (Russell & Gajos, 2020). Several studies demonstrated the validity of EMA to measure behavior of children with ADHD (Miguelez-Fernandez et al., 2018).

#### Attachment Representations

We used a story stem procedure to assess attachment representations (Bretherton et al., 1990). This is a narrative technique in which a trained interviewer starts to tell a story with dolls and props and the child is asked to finish the story, both verbally and using materials. The stories all have an attachment-related theme, and are meant to evoke secure base behaviors. The original task was used only in preschool children, but in this study we used the story stem tasks of Kerns’ lab (Kerns, 2016). For the younger children (4–8 years), we used the following stories: ‘Hurt knee’, ‘Monster in the bedroom’, ‘Separation from parent’, and ‘Reunion with parent’. For the older children (8–12 years), the first two stories were replaced by ‘School assistance’, and ‘Fight with friend’, to capture more age salient issues. The main character of each story stem represented the child him or herself, and the other doll represented the primary caregiver. All story stems were recorded, and coded independently by two raters (RH or research-assistants), who received a training in the assessment and scoring. All children received a score between 1 (no signs) and 5 (prototypical) on each pattern (secure, avoidant, ambivalent, disorganized), using the coding procedures developed by Kerns and colleagues (Kerns, 2016). Agreement between coding of attachment representations was very good for this sample (interrater reliability; *κ* = 0.82–0.96). Disagreements in ratings were discussed, and the final scores were based on unanimous decisions. Validity of the story stem procedure has been demonstrated, especially for secure and disorganized attachment (Kerns et al., 2011).

### Statistical Analyses

Data was analyzed on an intention-to-treat basis. ANOVA’s (continuous variables) and chi-squared tests (categorical variables) were used to assess differences between the three conditions in demographic and baseline characteristics, using the Statistical Package for the Social Science (SPSS, version 26). We conducted multilevel analyses (mixed models), which takes missing data into account (Twisk et al., 2013), using Stata, version 16 (StataCorp, 2019). We distinguished three levels: outcomes (level 1) nested in participants (level 2), nested in therapists (level 3). Random intercepts at therapist level were included if the Likelihood Ratio Test showed a significant improvement of the model fit. Power-analysis of an ANCOVA with baseline score as covariate and three groups revealed that we needed a total sample of 90 children, and thus 30 children per group, to detect intervention effects on our primary, proximal outcome (alpha = 0.05, power = 0.80, *df* = 1). To assess main effects of the condition, Condition (AC, CC, control) was included as between subjects factor and time (T1, T2) as within subjects variable. T0 scores were added as a fixed factor to control for baseline differences in our outcome measure. Second, moderator effects were assessed by examining interactions between the effect of condition and attachment representations (continuous). For each variable (secure, ambivalent, avoidant, disorganized), the condition by variable interaction was added to the model to assess whether the change over time differed between levels of the potential moderator. Effects were averaged over T1 and T2, provided that problem behaviors remained stable between T1 and T2. To address the first aim of our study, we assessed whether attachment representation moderated the effect on the primary outcome when comparing both active conditions together to the control condition (i.e., brief BPT versus waitlist). To address the second aim, we tested if attachment representation moderated the effect on the primary outcome when comparing the active conditions *with each other* (i.e., antecedent-based condition versus consequent-based condition). Since these moderator analyses were hypotheses-generating, correction for multiple testing was not applied (Bender & Lange, 2001; Rothman, 1990). We conducted additional analyses with IQ and age as covariates. In case of a moderating effect, we assessed if conditions were differentially effective for different levels of the moderator based on median‐split. The significance level for all analyses was set at *α* = 0.05, two-tailed. Effect sizes were computed by dividing the regression coefficient by the (pooled) standard deviation.

## Results

An overview of the demographic and baseline characteristics can be found in Table 1. Groups did not differ on these characteristics. For descriptive purposes, we provide classification frequencies of the attachment representations. Children were classified to the pattern for which they had the highest rating. In this sample 40 children (54%) were securely, 16 children (22%) were avoidant, 3 children (4%) were ambivalent, and 15 children (20%) were disorganized attached. Note that we used the continuous ratings in all analyses. Intercorrelations between the variables can be found in the Supplementary Information (Table A).

### Preliminary Analyses

In Supplementary Information, Table B, the effects of the intervention and the results of the moderator analyses are depicted. First, in line with previous findings in our larger sample Hornstra et al., 2021), we found a significant decrease in daily rated problem behaviors for both active conditions, compared to the control condition, with medium effect sizes (antecedent-based condition: *d* = 0.73, consequent-based condition: *d* = 0.56). The two active conditions did not differ from each other with regard to reduction in daily reported problem behaviors. Random intercepts at the ‘therapists’ level did not improve the model, therefore all models were reduced to two levels (observations clustered in children). Problem behaviors remained stable from T1 (i.e., post-intervention) to T2 (i.e., three week follow-up) within all conditions.

### Aim 1: Moderation of Attachment Representation on Brief BPT

None of the attachment representations (secure, avoidant, ambivalent, disorganized) moderated treatment outcomes when training (both antecedent- and consequent based techniques) was compared to the waitlist control condition (see Supplementary Information, Table B for an overview of all analyses).

### Aim 2: Moderation of Attachment Representation on Specific Techniques

#### Secure Attachment Representation

**Fig. 1:**
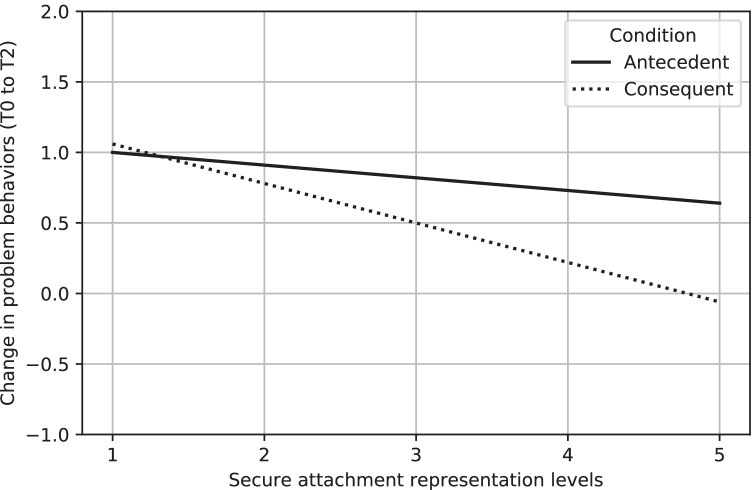
Interaction between the change in problem behaviors (y-axis, between T0 to T2; positive values indicate improved behavior, negative values indicate deteriorated behavior), and secure attachment representation levels (x-axis)

There was a significant interaction between secure attachment and intervention condition (antecedent-based versus consequent-based techniques; *B* = -0.27, *SE* = 0.12, *p* = 0.027, *d* = 0.28), depicted in Fig. 1. In the consequent-based condition, compared to the control condition, less securely attached children benefited more from the training than more securely attached children (*B* = 0.33, *SE* = 0.13, *p* = 0.01, *d* = -0.34). For the antecedent-based condition, compared to the control condition, these effects were not found (*B* = 0.05, *SE* = 0.12, *p* = 0.68, *d* = -0.05. Analyses showed similar patterns with age and IQ as covariates (Supplementary Information, Table C).

**Fig. 2:**
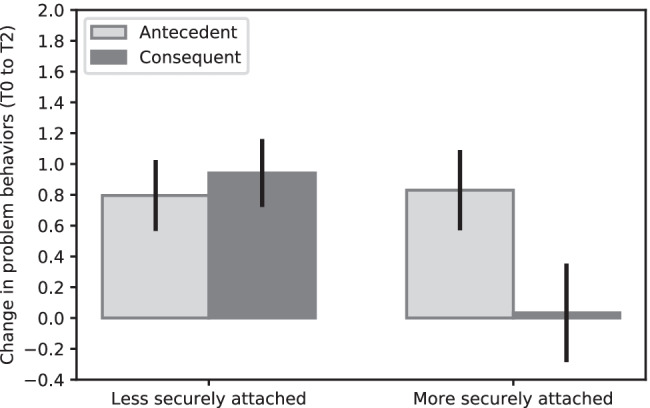
Change in problem behaviors between T0 and T2, organized by less securely attached children (secure attachment representation ≤ 2.75), and more securely attached children (secure attachment representation > 2.75), positive values indicate improved behavior, negative values indicate deteriorated behavior. Error bars depict one standard error

Follow-up analyses (shown in Fig. 2) based on median-split showed that for less securely attached children (secure attachment representation ≤ 2.75), there were no differences in effectiveness between the antecedent-based condition and the consequent-based condition (*B* = 0.24, *SE* = 0.18, *p* = 0.182, *d* = -0.24). For more securely attached children (secure attachment representation > 2.75), the effectiveness of the antecedent-based condition significantly differed from the consequent-based condition (*B* = -0.59, *SE* = 0.24, *p* = 0.014, *d* = 0.60). More securely attached children benefited more from the antecedent-based techniques than from the consequent-based techniques.

#### Avoidant Attachment Representation

No significant interaction between avoidant attachment representation and intervention condition (antecedent versus consequent) was found (*B* = 0.13, *SE* = 0.17, *p* = 0.442, *d* = -0.14).

#### Ambivalent Attachment Representation

Similarly, there was no interaction between ambivalent attachment representation and intervention condition (antecedent versus consequent) of the children (*B* = 0.28, *SE* = 0.24, *p* = 0.242, *d* = -0.28).

#### Disorganized Attachment Representation

**Fig. 3:**
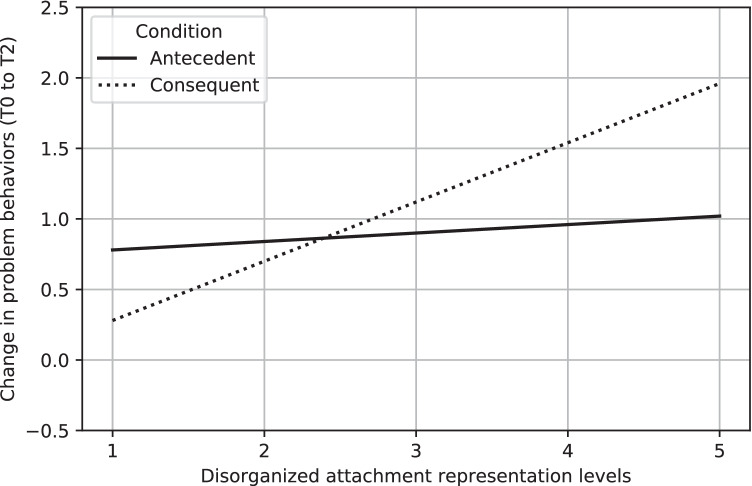
Interaction between the change in problem behaviors (y-axis, between T0 to T2; positive values indicate improved behavior, negative values indicate deteriorated behavior), and disorganized attachment representation levels (x-axis)

There was a significant interaction between disorganized attachment and intervention condition (antecedent-based versus consequent-based techniques; *B* = 0.36, *SE* = 0.16, *p* = 0.02, *d* = -0.36), shown in Fig. 3. Disorganized attachment did not significantly moderate the effects of the antecedent-based condition compared to the control condition (*B* = 0.06, *SE* = 0.13, *p* = 0.65, *d* = -0.06), nor the consequent-based condition compared to the control condition (*B* = -0.30, *SE* = 0.16, *p* = 0.07, *d* = 0.30). Analyses showed similar patterns with age and IQ as covariates (Supplementary Information, Table C).

**Fig. 4:**
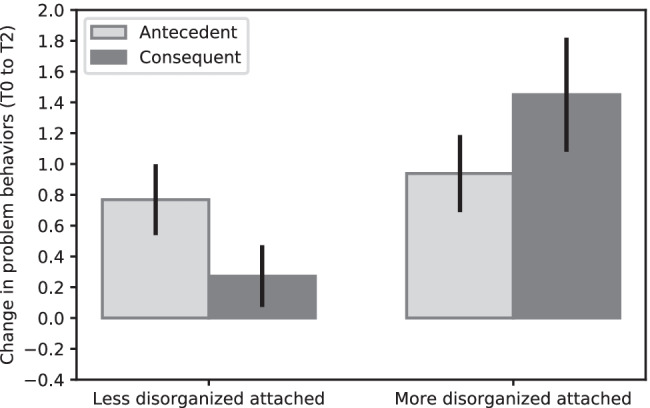
Change in problem behaviors between T0 and T2, organized by less disorganized attached children (disorganized attachment representation ≤ 2.00), and more disorganized attached children (secure attachment representation > 2.00), positive values indicate improved behavior, negative values indicate deteriorated behavior. Error bars depict one standard error

Follow-up analyses (depicted in Fig. 4) based on median-split showed that, for the group of less disorganized children (disorganized attachment representation ≤ 2.00), there were differences in effectiveness between the antecedent-based condition and the consequent-based condition (*B* = -0.45, *SE* = 0.18, *p* = 0.013, *d* = 0.46). Antecedent-based techniques were more effective than consequent-based techniques for less disorganized children. For more disorganized children (disorganized attachment representation > 2.00), the antecedent-based and the consequent-based condition also differed from each other (*B* = 0.59, *SE* = 0.23, *p* = 0.011, *d* = -0.59). Consequent-based techniques were more effective than antecedent-based techniques in decreasing problem behaviors for the more disorganized attached children.

## Discussion

This was the first study into the moderating effects of attachment of children with ADHD on the effects of BPT. We aimed (1) to explore whether attachment representations of children with ADHD were related to the effectiveness of brief BPT and (2) to explore whether attachment representations differentially affected the effects of either antecedent-based techniques or consequent-based techniques within this brief BPT program. First, we found that attachment representations were not associated with the effectiveness of brief BPT in general. Second, we showed that the effects of the two types of behavioral techniques used in the brief training programs differed between subgroups of children with ADHD. More securely attached children, and less disorganized attached children benefited less from the consequent-based techniques than from the antecedent-based techniques. More insecurely attached children benefited from both types of techniques, whilst children with a more disorganized attachment representation benefited more from the consequent-based techniques than from the antecedent-based techniques. If replicated, our findings may have important implications for clinical practice.

Regarding the first aim, we tentatively reasoned that the brief BPT program (regardless of specific techniques) was more effective for securely attached children, and less effective for children with an avoidant, anxious, of disorganized attachment representation. However, in contrast to the more general findings with regard to the link between insecure attachment and less favorable treatment outcomes (Levy et al., 2018; Slade & Holmes, 2019), we did not find this. Training parents in behavioral techniques was effective, also for children with a more insecure attachment representation. Apparently, even insecurely attached children notice and respond to changes in parenting strategies from their caregivers. Children are inherently inclined to seek support from their parents, and improved parenting practices seem to be valuable and effective, also for insecurely attached children (Bakermans-Kranenburg et al., 2003).

Regarding the second aim, we tentatively hypothesized that for insecurely and disorganized attached children the consequent-based techniques could be less effective, while the antecedent-based techniques could be effective regardless of attachment representation. We speculated that insecurely attached children could have difficulties interpreting rewarding strategies as positive, due to a lack of trust in their parents. However, with regard to the consequent-based techniques we found the opposite: Less securely attached children benefited from parental reinforcement and discipline strategies than more securely attached children, and more disorganized attached children benefited even more from these types of parenting techniques. Training the parents of less disorganized attached children in consequent-based techniques had less of an effect on the daily rated problem behaviors. Antecedent interventions such as avoiding conflict through clear rules and routines were beneficial regardless of attachment representation of the children. Here, we propose two possible explanations for our findings with regard to the consequent-based techniques.

First, it can be argued that consequent-based techniques (e.g., positive attention, other rewarding strategies) can be viewed as more sensitive and responsive, and thus attachment-related, than the somewhat neutral antecedent-based techniques (e.g., stating clear rules and instructions, providing routines). According to attachment theory, warm and responsive behaviors of caregivers (i.e., sensitive parenting) are thought to lead to a secure internal working model (Bowlby, 1969). Meta-analytic and empirical evidence confirms that parental sensitivity is an important predictor of attachment security (Beijersbergen et al., 2012; De Wolff & van Ijzendoorn, 1997). Consequent-based techniques are thought to boost the parent–child relationship, and therefore also promote a more secure relationship between the parent and the child (Allen et al., 2014; Thomas et al., 2017). It may be that parents of the securely and less disorganized attached children already used these types of techniques, therefore leaving less room for improvement. Hence, training these parents in specific consequent-based techniques did not change their parenting that much, and did not significantly improve the problem behaviors of their children. Unfortunately, we did not have information about the support-related practices of these caregivers before the start of the training, and therefore we cannot know if this was truly the case.

Another possible mechanism underlying our findings with regard to the consequent-based techniques can be derived from the differential susceptibility theory (Ellis et al., 2011). This theory proposes that susceptible individuals are more affected, for better and for worse, by their environment than their less susceptible counterparts. Observational and experimental research confirms that some children are especially susceptible to negative, but also positive environmental effects (see for a review Belsky, 2013). For example, in a study on a social-learning and attachment-based intervention aimed at increasing sensitive parenting and improving discipline strategies (i.e., consequent-based techniques), highly reactive children were most susceptible to the intervention and profited the most (Velderman et al., 2006). Based on these assumptions, it could be that the children with ADHD and higher levels of disorganization and attachment insecurity belong to a more susceptible subgroup of children and thus benefit more from the improved use of consequent-based techniques by their parents than their more securely attached peers. It is possible that this specific subgroup of children, due to the specific causal processes underlying ADHD, had problems signaling and learning from sensitive parenting practices while growing up, and therefore benefited from this brief training in consequent-based techniques. For example, altered reward and punishment sensitivity can result in difficulties learning from stimulus–response associations, and therefore more explicit and powerful reinforcement is needed to learn new adaptive behaviors (Tripp & Wickens, 2008; Van der Oord & Tripp, 2020).

### Strengths and Limitations

This was the first study to examine the influence of attachment representations on the effectiveness of behavioral techniques in a clinical sample of children with ADHD. A major strength of the study was the use of a story stem procedure to measure attachment representations, instead of other procedures such as self-reported questionnaires or observational methods. Representational measures such as the story stem procedure take possible biases such as social desirability, and the positive illusory bias of children with ADHD (i.e., the tendency to overestimate one’s own capacities; Volz-Sidiropoulou et al., 2016) into account. Given the broad age group of our study (4–12 years), the story stem procedure was an excellent option to measure attachment representations (Bosmans & Kerns, 2015), and seems to be more suitable than questionnaires for children with ADHD (Hornstra et al., 2019). The story stem procedure focused on the primary caregiver in the stories, and outcome was rated by the same primary caregiver. Therefore, the informant for the outcome measure and the person of focus with regard to the attachment representation match. Nonetheless, the current study should be interpreted with some limitations in mind. First, as mentioned above, we did not have a baseline measurement of parenting, including behaviors associated with child attachment (e.g., parental responsivity/sensitivity). Future studies could add this to gain more insight into the mechanism underlying the differential effects of BPT techniques. Second, parents received the training, but also rated our primary outcome. Therefore, an overestimation of the rated effects is a possible limitation (Daley et al., 2014). However, the use of EMA improves the ecological validity of the current study, as it reduces potential recall or memory bias (Russell & Gajos, 2020). Third, the follow up period was relatively brief (i.e., three weeks). Thus, it is not clear if moderative results extend to longer term effects of our intervention. Fourth, our sample was not explicitly selected on the base of insecure attachment representations, and approximately 50% of the children included in this study were securely attached. This could limit the generalizability of our findings to other, more vulnerable populations. Still, the percentage of children with an insecure or disorganized attachment representation was significantly and considerably higher than in typically developing children (Dekkers et al., 2021b), emphasizing the importance of attachment representations of children with ADHD as a potential moderator of treatment success. Fifth, the consequent-based techniques that were used for the individualized intervention plans could include a range of different techniques (i.e., planned ignoring, praise, rewards, and punishment). Potentially, certain consequent-based techniques, for example planned ignoring (a negative punishment technique) versus rewards, could be differentially effective for children (Yee & Shiota, 2015). Further research has to be conducted to examine exactly which techniques are most helpful and whether there is a differential effect of certain types of techniques for subgroups of children based on attachment representation, for example, punishment versus reward-based techniques. Sixth, because we assessed attachment representations of the children in the current sample at baseline, it remains unknown whether attachment changed following our brief BPT. Standard treatment strategies that target ADHD-symptoms seem to not in itself improve attachment security (Darling Rasmussen et al., 2021). The learning theory of attachment can help integrate attachment theory into clinical practice (Bosmans et al., 2020). Attachment based elements can strengthen regular antecedent- and consequent-based techniques in BPT (Bosmans et al., 2022). Evidence from parenting interventions in young children that specifically focus on parental sensitivity and the parent–child relation (e.g., Parent–Child Interaction Therapy [PCIT], Video-feedback Intervention to promote Positive Parenting and Sensitive Discipline [VIPP-SD]) show a beneficial effect of parenting programs on children’s attachment (Allen et al., 2014; Bakermans-Kranenburg et al., 2003; Juffer et al., 2017). This is in line with findings that children’s trust in caregivers can catch up due to improved parenting experiences (Bosmans et al., 2019; Verhees et al., 2020). However, in the current study, we did not include relationship building components to our training, which may have influenced our findings regarding the moderative effects of attachment representation. Relatedly, in the current study we assessed attachment representations towards the primary caregiver. In a minority of the cases, there is a discordance between attachment between both caregivers (Dagan & Sagi-Schwartz, 2018; Di Folco et al., 2020), and these discordant attachment representations can influence differential developmental outcomes (Dagan et al., 2022). Assessment of the attachment relationships towards both caregivers may provide a more complete picture of the influence of attachment on behavioral strategies. Seventh, in the current study we found an influence of attachment representations on the effectiveness behavioral techniques in a clinical sample of children with ADHD. Nevertheless, the question remains whether this is a unique effect within children with ADHD or a more general effect of attachment representations in broader samples of children with behavioral problems. Eighth, the current study was not a priori powered for moderation analyses. Our power-analysis was based on detection of differences between the intervention conditions and control condition on our outcome measure. Therefore, although generally microtrials can have greater power to detect moderation with smaller sample sizes (Howe et al., 2010; Howe & Ridenour, 2019), power to detect possible moderation effects in this study may have been too low, possibly leading to small effects remaining undetected. Lastly, it remains unknown which processes and mechanisms of change are involved in the differential effects of behavioral techniques, and future studies on the current topic, also in other samples, are therefore required (Kazdin, 2007).

## Conclusion

This study highlights the importance of studying attachment as potential moderator of behavioral strategies used by parents of children with ADHD. The results of our study tentatively suggest that in BPT for children with ADHD, behavioral parent training techniques are differentially effective based on attachment representation. Replication of our findings is warranted, as more knowledge about moderating effects may guide future treatment development and tailoring.

## Supplementary Information

Below is the link to the electronic supplementary material.

Supplementary file1 (DOCX 17 KB)

## Data Availability

The data are not publicly available because participating families did not consent for this.
