# Supplementary material for: Attachment Representation Moderates the Effectiveness of Behavioral Parent Training Techniques for Children with ADHD: Evidence from a Randomized Controlled Microtrial
Source: Res Child Adolesc Psychopathol. 2022 Apr 1;50(9):1151–64. doi: 10.1007/s10802-022-00921-5 (PMC9525431; doi:10.1007/s10802-022-00921-5)
Supplement: Supplementary file 1 — Supplementary file1 (DOCX 17 KB) [file 10802_2022_921_MOESM1_ESM.docx]

**Table A**. Pearson correlations between all variables

|  | 1. | 2. | 3. | 4. | 5. | 6. | 7. | 8. | 9. | 10. | 11. | 12. | 13. | 14. |
| --- | --- | --- | --- | --- | --- | --- | --- | --- | --- | --- | --- | --- | --- | --- |
| 1. Secure | - | -.500** | -.250* | -.699** | .190 | .199 | .001 | -.183 | -.041 | -.160 | .061 | .057 | -.144 | -.147 |
| 2. Avoidant |  | - | -.255* | -.062 | .035 | .026 | .133 | .074 | -.209 | -.125 | -.038 | .037 | .142 | .135 |
| 3. Ambivalent |  |  | - | .301** | -.105 | .000 | .075 | .113 | .072 | .134 | -.086 | -.149 | .003 | -.099 |
| 4. Disorganized |  |  |  | - | -.287* | -.223 | -.074 | .117 | .294* | .324** | -.002 | -.044 | .103 | .127 |
| 5. Age |  |  |  |  | - | .207 | .270* | .152 | -.379** | -.228 | -.107 | -.085 | -.113 | -.080 |
| 6. IQ |  |  |  |  |  | - | .294* | .011 | -.070 | -.029 | .065 | .033 | .025 | .038 |
| 7. Parental educational level  ^a^ |  |  |  |  |  |  | - | .060 | -.141 | -.072 | -.103 | -.100 | .092 | .117 |
| 8. Inattention symptoms  ^b^ |  |  |  |  |  |  |  | - | -.084 | .540** | -.056 | .223 | -.164 | -.067 |
| 9. Hyperactivity-impulsivity symptoms  ^b^ |  |  |  |  |  |  |  |  | - | .788** | .161 | .139 | .197 | .156 |
| 10. ADHD symptoms ^b^ |  |  |  |  |  |  |  |  |  | - | .115 | .256* | .055 | .085 |
| 11. ODD symptoms  ^b^ |  |  |  |  |  |  |  |  |  |  | - | .394** | .296* | .342** |
| 12. CD symptoms ^b^ |  |  |  |  |  |  |  |  |  |  |  | - | .143 | .186 |
| 13. Impairment (number of domains)  ^c^ |  |  |  |  |  |  |  |  |  |  |  |  | - | .886** |
| 14. Impairment (average score)  ^c^ |  |  |  |  |  |  |  |  |  |  |  |  |  | - |

*Note. N* = 74. * *p* < .05, ***p* < .01, ****p* < .001. ADHD = attention-deficit/hyperactivity disorder; ODD = oppositional defiant disorder; CD = conduct disorder. ^a^ Parental education level (average of both parents) was classified according to the Dutch classification system: 1=no education completed, 2=early childhood education, 3=primary education, 4=lower secondary education, 5=upper secondary education, 6=undergraduate school, 7=graduate school, 8=post-graduate education, which was divided in low=1, 2, 3, 4, medium=5, and high=6, 7, 8 (CBS, 2006).

^b^ Assessed with the Diagnostic Interview Schedule for Children-IV-TR, adapted to the DSM-5. ^c^ Assessed with the Impairment Rating Scale; domains with a score >3 were classified as impaired.

**Table B.** Intervention effects and results of the moderator analysis (intervention condition by variable interaction)

|  | BPT vs. WL | | | AC vs. CC | | | AC vs. WL | | | CC vs. WL | | |
| --- | --- | --- | --- | --- | --- | --- | --- | --- | --- | --- | --- | --- |
| Daily rated problem behaviors | *B (SE)* | *p* | *d* | *B (SE)* | *p* | *d* | *B (SE)* | *p* | *d* | *B (SE)* | *p* | *d* |
|  | -.64 (.13) | **<.001** | **.63** | -.17 (.15) | .269 | .17 | -.72 (.15) | **<.001** | **.73** | -.55 (.15) | **<.001** | **.56** |
| Moderator analyses | | | | | | | | | | | | |
| Secure | .17 (.11) | .12 | -.17 | -.27 (.12) | **.03** | **.28** | .05 (.12) | .68 | -.05 | .33 (.13) | **.01** | **-.34** |
| Avoidant | -.27 (.15) | .07 | .27 | .13 (.23) | .57 | -.14 | -.22 (.22) | .33 | .22 | -.35 (.22) | .12 | .36 |
| Ambivalent | .11 (.22) | .61 | -.11 | .28 (.31) | .37 | -.28 | .22 (.24) | .36 | -.22 | -.06 (.26) | .82 | .06 |
| Disorganized | -.06 (.12) | .68 | .05 | .36 (.16) | **.02** | **-.36** | .06 (.13) | .65 | -.06 | -.30 (.16) | .07 | .30 |

*Note. N* = 74. AC = Antecedent-based condition; BPT = Behavioral Parent Training; CC = Consequent-based Condition; WL = waitlist.

**Table C.** Results of the moderator analysis with IQ and age as covariate (intervention condition by variable interaction)

|  | BPT vs. WL | | | AC vs. CC | | | AC vs. WL | | | CC vs. WL | | |
| --- | --- | --- | --- | --- | --- | --- | --- | --- | --- | --- | --- | --- |
| Moderator analyses with IQ and age as covariate | | | | | | | | | | | | |
| Secure | .17 (.11) | .11 | -.17 | -.24 (.12) | **.04** | **.24** | .07 (.12) | .55 | -.07 | .32 (.12) | **.01** | **-.33** |
| Avoidant | -.22 (.15) | .14 | .22 | .14 (.18) | .43 | -.14 | -.16 (.17) | .34 | .16 | -.31 (.17) | .08 | .32 |
| Ambivalent | .13 (.22) | .54 | -.13 | .21 (.23) | .36 | -.21 | .19 (.24) | .42 | -.19 | -.02 (.26) | .93 | .02 |
| Disorganized | -.10 (.12) | .41 | .10 | .34 (.16) | **.03** | **-.35** | -.00 (.12) | .99 | .00 | -.34 (.16) | **.04** | **.35** |

*Note. N* = 74. AC = Antecedent-based condition; BPT = Behavioral Parent Training; CC = Consequent-based Condition; WL = waitlist.
